# Supplementary material for: Assessing Chronodisruption Distress in Goldfish: The Importance of Multimodal Approaches
Source: Animals (Basel). 2023 Aug 1;13(15):2481. doi: 10.3390/ani13152481 (PMC10417125; doi:10.3390/ani13152481)
Supplement: Supplementary file 1 [file animals-13-02481-s001.zip › Table S1- Editable.pdf]

Gene	Access number (GenBank)	Sequence (5'→3')		Product (bp)
β-actin	AB039726.2	Forward	CAGGGAGTGATGGTTGGCA	168
		Reverse	AACACGCAGCTCGTTGTAGA	
ef-1α	AJ431209	Forward	CCCTGGCCACAGAGATTTCA	101
		Reverse	CAGCCTCGAACTCACCAACA	
crf	AF098629	Forward	GGCTCTGCTCGTTGCCTTT	121
		Reverse	CCCTAAGCGTGCCAAAACC	
pomc	AJ431209	Forward	CTCACCACTGACGAGAACATCTTG	161
		Reverse	CGGTTTGCTCCAGCTCAGA	
