# Supplementary material for: Assessing Chronodisruption Distress in Goldfish: The Importance of Multimodal Approaches
Source: Animals (Basel). 2023 Aug 1;13(15):2481. doi: 10.3390/ani13152481 (PMC10417125; doi:10.3390/ani13152481)
Supplement: Supplementary file 1 [file animals-13-02481-s001.zip › Table S2- Editable.pdf]

		Open field with object approach				
		Latency to open field	Entries to open field	Time in open field	Latency to object	Average velocity
Black/white preference test	Latency to white	P = 0.68<br>CC = -0.06	P = 0.38<br>CC = 0.13	P = 0.69<br>CC = 0.06	P = 0.19<br>CC = 0.34	P = 0.513<br>CC = 0.09
	Crossings between zones	P = 0.66<br>CC = -0.06	P = 0.43<br>CC = 0.11	P = 0.22<br>CC = -0.18	P = 0.77<br>CC = -0.04	P = 0.01<br>CC = 0.35
	Time in white	P = 0.98<br>CC = 0.003	P = 0.66<br>CC = 0.06	P = 0.74<br>CC = -0.05	P = 0.6<br>CC = -0.08	P = 0.14<br>CC = 0.23
	Average velocity	P = 0.17<br>CC = -0.19	P = 0.11<br>CC = 0.23	P = 0.31<br>CC = -0.15	P = 0.54<br>CC = -0.11	P = 0.007<br>CC = 0.39

Table S2. Spearman correlations between behavioral parameters of the black/white and open field tests performed by the same fish, the same day. $p < 0.05$ (green) indicates significant relationship between the two variables ($n = 48$). CC= correlation coefficient.
